# The Reduction Maneuver for Pediatric Extension Type 3 Supracondylar Humerus Fractures

**DOI:** 10.7759/cureus.9213

**Published:** 2020-07-15

**Authors:** Dallas Smuin, Mark Hatch, Zachary Winthrop, Sandeep Gidvani, William Hennrikus

**Affiliations:** 1 Orthopaedics, Penn State Health Milton S. Hershey Medical Center, Hershey, USA; 2 Orthopaedics, Rosenberg Cooley Metcalf Orthopedic Clinic, Park City, USA; 3 Pediatrics, Boston Children's Hospital, Boston, USA; 4 Orthopaedics, OrthoNorCal, Inc., Los Gatos, USA

**Keywords:** supracondylar humeral fracture, pediatric fractures

## Abstract

Extension type supracondylar humerus fractures in children commonly displace in two directions: posteromedial and posterolateral. The traditional maneuver to reduce posteromedial displaced fractures utilizes pronation of the forearm, while the maneuver for posterolateral displaced fractures utilizes supination. Traditional teaching suggests that the periosteum is an aid to reduction. The purpose of this study is to take a second look at this periosteal hinge theory and reexamine the maneuver performed when reducing an extension type 3 supracondylar fracture. Sixty-nine consecutive displaced extension type 3 supracondylar fractures were studied. Intraoperative fluoroscopic radiographs were graded as posteromedial, posterolateral, or direct posterior displacement. All fractures were treated with closed reduction and percutaneous pinning. The best maneuver used to align the fracture during surgery was recorded in the operative note. The direction of displacement on radiographs was 32 (46.3%) posteromedial, 31 (45%) posterolateral, and six (8.7%) direct posterior. All of the 32 posteromedial displaced fractures were best aligned when pronation was utilized. All of the 31 posterolaterally displaced fractures were best aligned when supination was utilized. The six direct posteriorly displaced fractures obtained the best alignment in pronation. The current study reaffirms the classic teaching that the direction of displacement of the fracture indicates the site of the intact periosteum. The intact periosteal hinge can be used to obtain fracture reduction.

## Introduction

Most supracondylar fractures result from a hyperextension force at the elbow when a child falls on the outstretched hand. The olecranon acts as a fulcrum, displacing the distal humeral fragment posteriorly, resulting in an extension supracondylar fracture [1-2]. Completed displaced extension supracondylar fractures are classified as Gartland type 3 [3]. Classic teaching suggests that Gartland type 3 extension-type pediatric supracondylar fractures disrupt the periosteum anteriorly. Posteriorly, the periosteum remains intact and can be used as a hinge to aid in reducing the fracture [4-5].

In 1974, Rang published his observations about the value of the periosteal hinge in the closed reduction of displaced supracondylar fractures [6]. He stated that when there was posteromedial displacement, a posteromedial periosteal hinge was always present and that pronation of the forearm tightened this medial hinge. He also proposed that for the fractures displaced posterolaterally, a posterolateral periosteal hinge was present and the fracture was best reduced by supination.

Rang traced the value of the periosteal hinge to Smith in 1894. Smith published findings on artificially produced supracondylar fractures on adult elbow cadaver specimens and concluded that elbow pronation and flexion stabilized the fracture reduction [5]. However, in 1982, Abraham challenged the periosteal hinge theory [7]. Using a monkey cadaver study, Abraham demonstrated that the periosteum had no impact on the reduction of supracondylar fractures; rather, a medial bony hinge was present in both medial and laterally displaced fractures. This bony hinge and pronation only helped to reduce both posteromedially and posterolaterally displaced fractures.

The purpose of this study was to take a second look at this periosteal hinge theory and reexamine the maneuver performed when reducing a Gartland type 3 extension supracondylar fracture. 

## Materials and methods

The Penn State Health Institutional Review Board issued approval PRAMS040362EP for this study. An analysis of one senior surgeon’s (WH) cases was performed. Sixty-nine consecutive displaced extension type 3 supracondylar fractures were reviewed. The inclusion criterion was a closed extension Gartland type 3 supracondylar fracture, treatment by closed reduction and percutaneous pinning, and age < 13 years. Exclusion criteria were non-Gartland type 3 fractures and open fractures.

**Figure 1:**
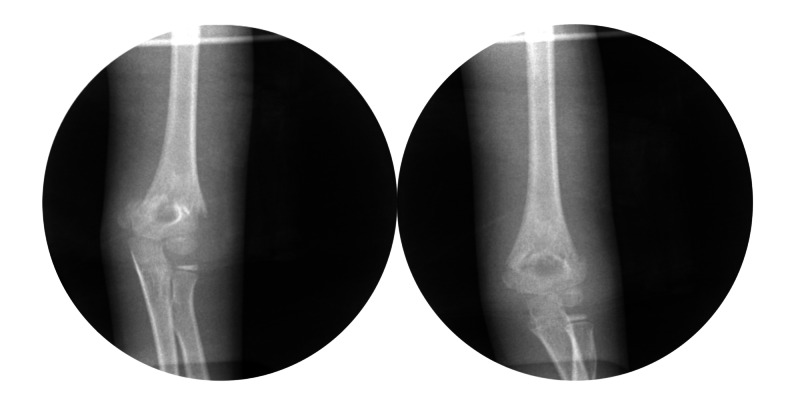
Intraoperative reduction of a posteromedial displaced supracondylar fracture The left image is the displaced fracture. The right image is the same fracture reduced with pronation.

**Figure 2:**
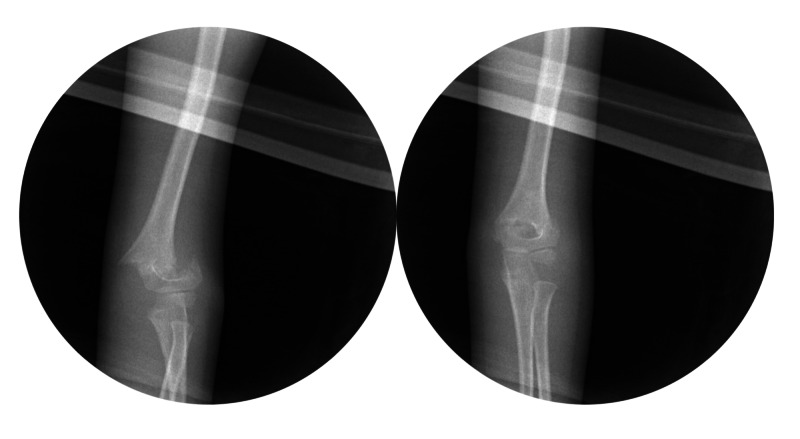
Intraoperative reduction of a posterolateral displaced supracondylar fracture The left image is the displaced fracture. The right image is the same fracture reduced with supination.

The reduction maneuver utilized to obtain a reduction of the fracture was recorded by the attending in the operative note. Fluoroscopic images were saved, demonstrating the best reduction maneuver (Figures 1, 2). After bringing the fracture to length with traction in extension, the forearm was rotated into pronation and then supination; the reduction of the humeral columns were compared under fluoroscopy. The best alignment--either pronation or supination--was chosen, and the elbow was then flexed to 130° and the reduction checked with fluoroscopy on the lateral view. Percutaneous pinning was then performed. Fixation was performed with two or three smooth 2 mm smooth Kirshner pins [8]. The elbow was extended, and anteroposterior (AP) and lateral fluoroscopic pictures were taken; the stability of fixation was evaluated with continuous fluoroscopy for a few seconds. The fracture was then cast with the elbow at 75° of flexion, and the cast was bi-valved to allow for swelling. Additional data analyzed included age, gender, arm injured, and average surgical time.

## Results

Sixty-nine fractures in 37 boys and 32 girls were studied. The average age was 6.2 years old (range: 2 - 12.4). Thirty-nine left elbow and 30 right elbows were injured. The average surgical time was 25 minutes (range: 9 - 48). No fracture was indicated for an open reduction. The direction of displacement on intraoperative fluoroscopic images was 32 (46.3%) posteromedial, 31 (45%) posterolateral, and six (8.7%) direct posterior. All of the 32 posteromedial displaced fractures were best aligned with pronation. All of the 31 posterolaterally displaced fractures were best aligned with supination The six direct posteriorly displaced fractures obtained the best alignment in pronation (Table 1).

**Table 1:** Patient Demographics Are Documented With Pertinent Findings Statistically significant findings were not observed.

	Male	Female	Average Age (Years)	Left Arm	Right Arm	Average Surgical Time (Minutes)	Open Reduction Required
Posteromedial	20	12	6.4	23	9	25	0
Posterolateral	14	17	6.1	16	15	28	0
Direct Posterior	3	3	5.8	4	2	18	0

## Discussion

The periosteum in the pediatric population is thicker and stronger than that of an adult [1, 9]. When a pediatric fracture occurs, the periosteum usually remains intact on the compression side of the injury and tears on the extension side [1]. The intact periosteum acts as a periosteal hinge [1, 10-11]. The hinge can contribute to the intrinsic stability of the fracture and aid in closed fracture reduction in children.

In the setting of a displaced extension type supracondylar fracture, Rang and Smith observed that a posteromedially displaced fracture could be reduced with pronation of the forearm and flexion of the elbow; pronation tightened the medial periosteal hinge [4-5]. Conversely, Abraham et al. challenged this periosteal hinge theory and stated that a medial bony hinge, and not the periosteum, was responsible for aiding the reduction of all extension supracondylar fractures [7].

**Figure 3:**
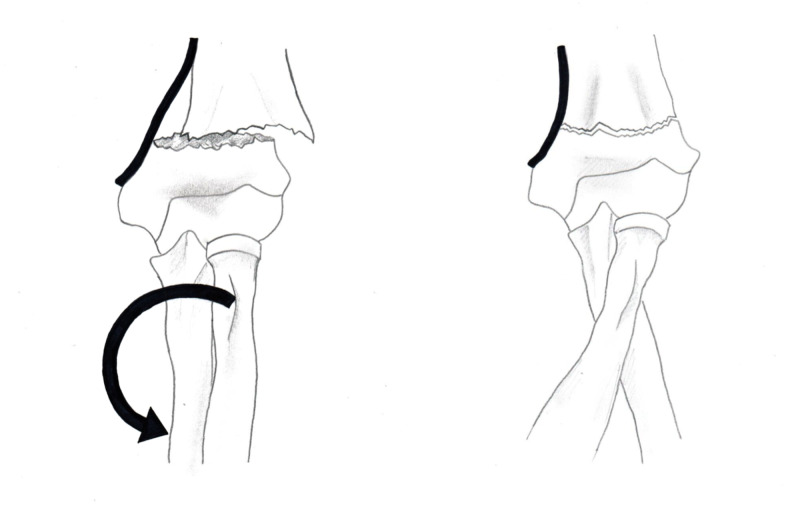
Posteromedial displaced supracondylar fracture The images demonstrate the effect of pronation tightening the periosteum and improving fracture alignment. (The bold line is demonstrated as periosteum in this image.)

**Figure 4:**
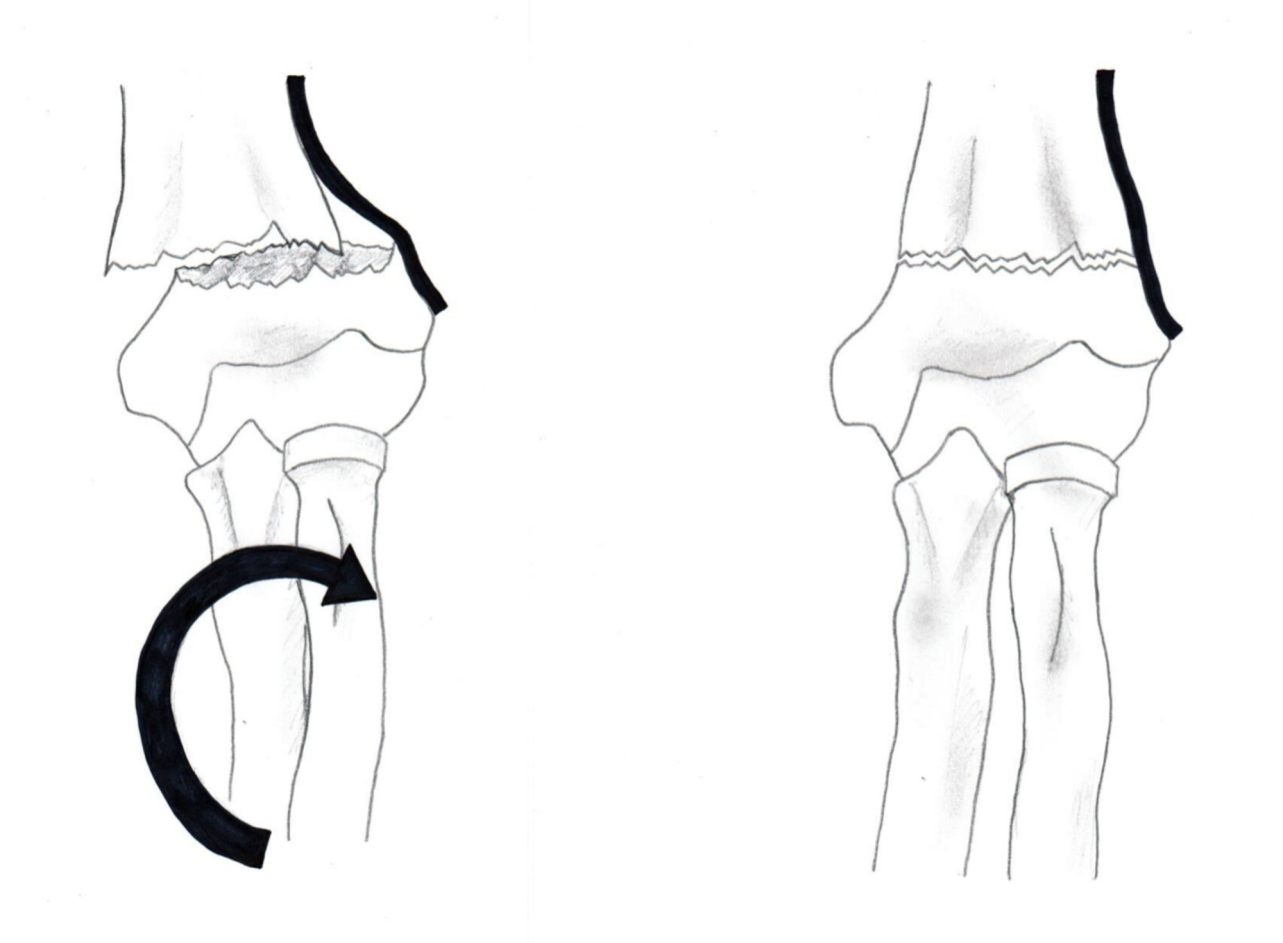
Posterolateral displaced supracondylar fracture The images demonstrate the effect of supination tightening the periosteum and improving fracture alignment. (The bold line is demonstrated as intact periosteum in this image)

The results of the current study support the principles proposed by Rang and Smith [4-5]. The periosteal hinge theory is valid. The reduction mechanism should be based on the direction of fracture displacement. The posteromedial displaced fractures obtained the best alignment with pronation and flexion; the posterolateral displaced fractures obtained the best alignment with supination and flexion (Figures 1-4). Understanding the direction of fracture displacement and location of intact periosteum helps the surgeon plan for and perform a successful reduction.

Limitations in the current study include all fractures were treated by a single surgeon at one center. The closed reduction did not allow for direct visualization of the periosteum.

## Conclusions

Pediatric fracture care is like a game of chess. The surgeon needs to anticipate the moves of each fracture piece and the intricate anatomy involved in the fracture. When treating pediatric supracondylar fractures, the use of the intact periosteal hinge to aid and maintain reduction is key. Our study reaffirms the classic teaching suggesting that the intact periosteum can be used for fracture reduction.
